
JOURNAL INFORMATION
==============================
NLM Title Abbreviation: CPT Pharmacometrics Syst Pharmacol
Journal ID: CPT Pharmacometrics Syst Pharmacol
NLM Journal ID: 101580011
Journal ID: PSP4

CPT: Pharmacometrics & Systems Pharmacology

EISSN: 2163-8306
Publisher: Wiley

ARTICLE INFORMATION
==============================
PMCID: PMC11474347
DOI: 10.1002/psp4.12984
Article ID: PSP412984
Article version: 1
Subjects: Issue Information, Issue Information

Issue Information

Electronic publication date: 2024 Jan 13
Publication date: 2024 Jan
Volume: 13
Issue: 1
First page: 1
Last page: 4
Copyright: © 2024 ASCPT
License: This is an open access article under the terms of the http://creativecommons.org/licenses/by/4.0/ License, which permits use, distribution and reproduction in any medium, provided the original work is properly cited.
License URL: https://creativecommons.org/licenses/by/4.0/

Figure count: 0
Table count: 0
Page count: 3

source-schema-version-number: 2.0
cover-date: January 2024
details-of-publishers-convertor: Converter:WILEY_ML3GV2_TO_JATSPMC version:6.4.9 mode:remove_FC converted:15.10.2024

==============================

An Official Journal of ASCPT and ISoP

### CPT: Pharmacometrics & Systems Pharmacology

Open Access

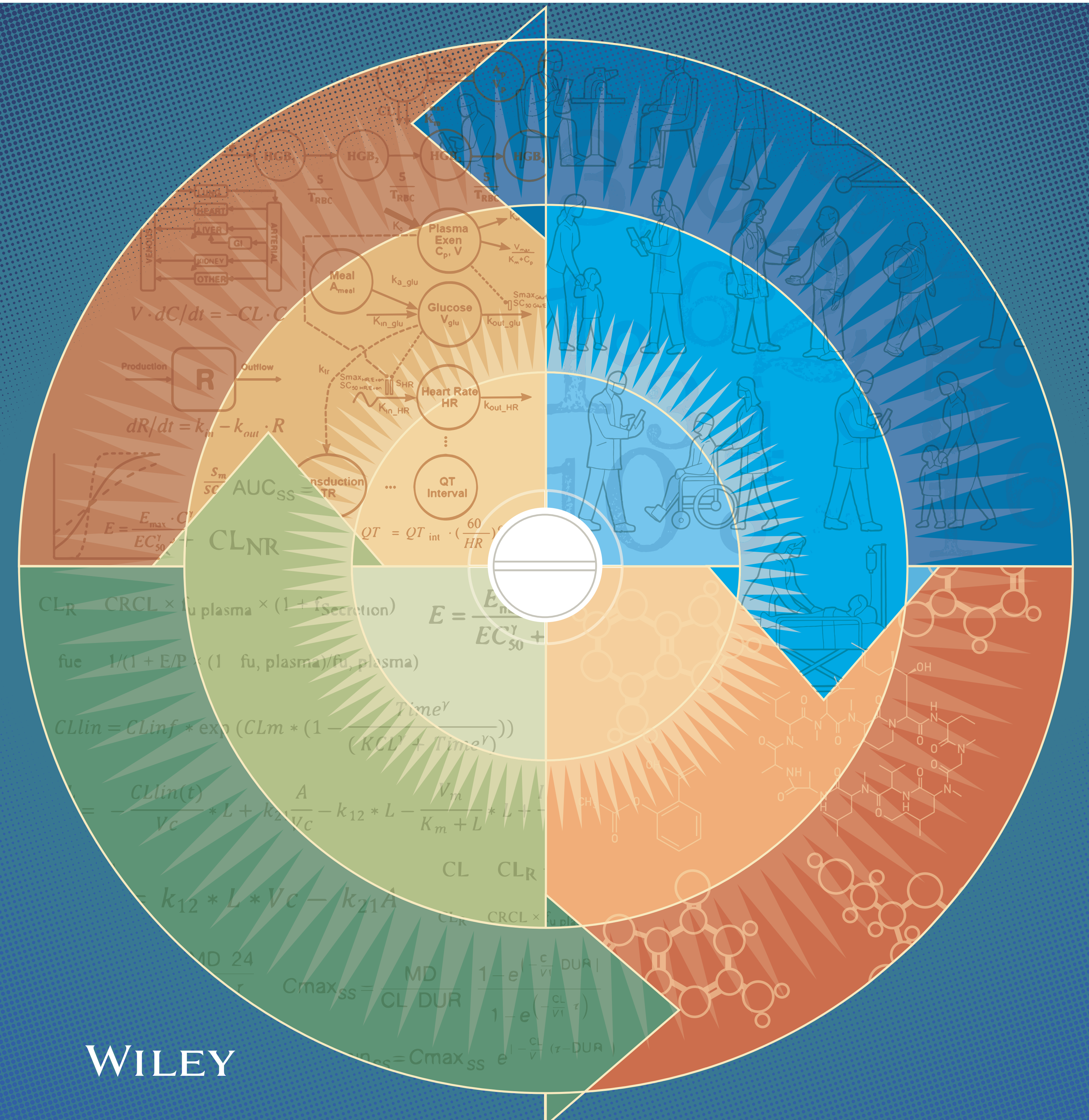

WILEY

### CPT: Pharmacometrics & Systems Pharmacology

WILEY

### Editor-in-Chief

France Mentré, MD, PhD, *School of Medicine of University Paris Diderot, Paris, France*

### Deputy Editor-in-Chief

Karen Rowland Yeo, PhD, *Certara UK Ltd., Sheffield, UK*

### Associate Editors

Paolo Denti, PhD, *University of Cape Town, Cape Town, South Africa*
Rajat Desikan, PhD, *GSK, Stevenage, UK*
Jonathan French, ScD, *Johnson & Johnson Innovative Medicine, Spring House, PA, USA*
Stefanie Hennig, MSc, PhD, *Certara Strategic Consulting, Brisbane, Australia*
Priya Jayachandran, PharmD, *Regeneron Pharmaceuticals, Inc., San Francisco, CA, USA*
Andrew Hooker, PhD, *Uppsala University, Uppsala, Sweden*
Sriram Krishnaswami, PhD, *Pfizer, Groton, CT, USA*
Ping Zhao, PhD, *Bill & Melinda Gates Foundation, Seattle, WA, USA*

### Editors-in-Training

Brian Cicali, PhD, *University of Florida, College of Pharmacy, Orlando, FL, USA*
Jane Knöchel, PhD, *AstraZeneca , Mölndal, Sweden*

### American Society for Clinical Pharmacology and Therapeutics

***Chief Executive Officer***
Abigail W. Gorman, *Alexandria, VA*

***Chief Operating Officer***
Elise S. Laffman-Johnson, *Alexandria, VA*

***Managing Editor***
Alethea B. Gerding, *Alexandria, VA*

***Deputy Managing Editor***
Alaina Webster, *Alexandria, VA*

***Senior Editorial Coordinator***
Aimee Greene, *Alexandria, VA*

### Editorial Board

Vivaswath S. Ayyar, PhD, *Janssen Research & Development, Spring House, PA*
Robert Bies, PharmD, PhD, *State University of New York at Buffalo, Buffalo, NY, USA*
Marc Birtwistle, PhD, *Clemson University Clemson, SC, USA*
Yuan Chen, PhD, *Genentech South San Francisco, CA, USA*
Marylore Chenel, PharmD, PhD, *Pharmetheus Uppsala, Sweden*
Emmanuel Chigutsa, PhD, *Eli Lilly and Company, Indianapolis, IN, USA*
Teresa Dalla Costa, PhD, *Federal University of Rio Grande do Sul - UFRGS Porto Alegre, Brazil*
Narendra M Dixit, PhD, *Indian Institute of Science, Bengaluru, India*
Anne-Gaelle Dosne, PharmD, PhD, *Janssen Research & Development, Beerse, Belgium*
Thomas Dumortier, Eng, MSc, *Novartis Pharma AG, Basel, Switzerland*
James M. Gallo, PharmD, PhD, *University at Buffalo, Buffalo, NY, USA*
Wei Gao, PhD, *EMD Serono, Billerica, MA, USA*
Jérémie Guedj, PhD, *INSERM, Paris, France*
Manish Gupta, PhD, FCP, *GenMab; Princeton, NJ, USA*
Bart Hendriks, PhD, *Novartis Institutes for BioMedical Research Cambridge, MA, USA*
Nick Holford, MBChB, *The University of Auckland Auckland, New Zealand*
Brett A. Howell, PhD, *DILIsym Services Division, Simulations Plus, Inc., Research Triangle Park, NC, USA*
Manuel Ibarra, PhD, *Universidad de la República, Montevideo, Uruguay*
Siv Jönsson, PhD, *Uppsala University Sweden, Uppsala, Sweden*
Kristin Karlsson, PhD, *Uppsala University Uppsala, Sweden*
Helen Kastrissios, PhD, *QuanTx Consulting, Brisbane, Australia*
Ron Keizer, PhD, *InsightRX San Francisco, CA, USA*
Steven E. Kern, PhD, *Global Health Labs, Bellevue, WA, USA*
Akash Khandelwal PhD, *UCB Biosciences GmbH, Monheim am Rhein, Germany*
Andrzej Kierzek, PhD, *Certara QSP; Sheffield, United Kingdom*
Qi Liu, PhD, *US Food and Drug Administration, Silver Spring, MD*
James Lu, PhD, *Genentech, Inc., South San Francisco, CA, USA*
Rajanikanth (Raj) Madabushi, PhD, *US Food and Drug Administration, Silver Spring, MD*
Efthymios Manolis, MSc, *European Medicines Agency London, United Kingdom*
Samer Mouksassi, PharmD, PhD, *Certara, Cairo, Egypt*
Jackson K. Mukonzo, PhD, *Makerere University, Kampala Uganda*
Joakim Nyberg, PhD, *Pharmetheus, Uppsala, Sweden*
Elsje Pienaar, PhD, *Weldon School of Biomedical Engineering, Purdue University, West Lafayette, IN, USA*
Daniel Scholes Rosenbloom, PhD, *Merck & Co., Rahway, NJ, USA*
Joe Standing, PhD, *University College London London, United Kingdom*
Natasha Strydom, PhD, *Amgen, Inc., San Francisco, CA, USA*
Yuichi Sugiyama, PhD, *Riken Innovation Center Sugiyama Laboratory Yokohama, Japan*
Elin Svensson, PhD, *Uppsala University, Uppsala, Sweden*
Joel Tarning, PhD, *Mahidol-Oxford Tropical Medicine Research Unit Bangkok, Thailand*
Nadia Terranova, PhD, *Merck KGaA, Lausanne, Switzerland*
Michael Tortorici, PharmD, PhD, *Aro Biotherapeutics, Philadelphia, PA, USA*
Coen van Hasselt, PhD, *Leiden University, Leiden, the Netherlands*
Paolo Vicini, PhD, MBA, *Confo Therapeutics, Ghent, Belgium*
Camille Vong, PhD, *Novartis AG Pharma, Basel, Switzerland*
Xinyuan (Susie) Zhang, PhD, *Daiichi Sankyo, Inc. Basking Ridge, NJ, USA*

### Overview

*CPT: Pharmacometrics & Systems Pharmacology* (PSP) is an official open access journal of the American Society for Clinical Pharmacology and Therapeutics (ASCPT) and International Society of Pharmacometrics (ISoP).

*CPT: Pharmacometrics & Systems Pharmacology* is a cross-disciplinary journal devoted to publishing advances in quantitative (e.g., modeling and simulation) methods as applied in pharmacology, physiology and therapeutics in humans. The journal welcomes original research articles, reviews and tutorials that bridge the following areas: pharmacometrics, modeling and simulation as applied to the design and evaluation of clinical trials, systems pharmacology modeling, particularly with a mechanistic link to human (patho)physiology, disease modeling, “population” or mixed-effects pharmacokinetics and pharmacodynamics (PKPD) modeling, modeling and simulation to support translational research, physiologically-based pharmacokinetics (PBPK), model-based meta-analyses of clinical trials, mechanism-based pharmacokinetic-pharmacodynamic modeling, computational pharmacology, bioinformatics, comparative efficacy, effectiveness and cost-effectiveness. Systems pharmacology may involve the application of systems biology approaches to study drug activities, targets and effects. The discipline is often defined with reference to engineering and pharmacological principles as the quantitative analysis of the dynamic interactions between drugs and a biologic system that aims to understand the behavior of the system as a whole. The common focus will be on quantitative methods that improve our understanding of pharmacology and therapeutics in humans.

**Abstracting and Indexing**
- Embase (Elsevier)
- SCOPUS (Elsevier)
- MEDLINE/PubMed indexing

**Society Information**
The American Society for Clinical Pharmacology and Therapeutics (ASCPT) was founded in 1900, and consists of over 2,200 professionals whose primary interest is to advance the science and practice of clinical pharmacology and translational medicine for the therapeutic benefit of patients and society. ASCPT is the largest scientific and professional organization serving the disciplines of Clinical Pharmacology and Translational Medicine.

**All articles published in this journal are freely available to read, download, and share. Articles are published under the terms of the Creative Commons Attribution Non-Commercial License which permits use, distribution, and reproduction in any medium, provided the original work is properly cited and is not used for commercial purposes.**

**Disclaimer**
The Publisher, the American Society for Clinical Pharmacology and Therapeutics, the International Society of Pharmacometrics, and Editors cannot be held responsible for any errors in or any consequences arising from the use of information contained in this journal. The views and opinions expressed do not necessarily reflect those of the Publisher, the American Society for Clinical Pharmacology and Therapeutics, the International Society of Pharmacometrics or Editors, neither does the publication of advertisements constitute any endorsement by the Publisher, the American Society for Clinical Pharmacology and Therapeutics, the International Society of Pharmacometrics, Editors, or Authors of the products advertised.

**Publisher**
*CPT: Pharmacometrics & Systems Pharmacology* is published by Wiley Periodicals, LLC, 101 Station Landing, Suite 300, Medford, MA 02155; Telephone: 781-388-8200; Fax: 781-388-8210.

CONTENTS

CPT: Pharmacometrics & Systems Pharmacology

### TUTORIAL

### TUTORIAL

5 A tutorial for model-based evaluation and translation of cardiovascular safety in preclinical trials
**Victoria Kulesh, Igor Vasyutin, Alina Volkova, Kirill Peskov, Holly Kimko, Victor Sokolov and Ravindra Alluri**

### RESEARCH

### CASE REPORT

23 Application of the model-informed drug development paradigm to datopotamab deruxtecan dose selection for late-stage development
**Yasong Lu, Shuang Liang, Ying Hong, Naoyuki Tajima, Kashyap Patel, Hanbin Li, David R. Wada, Jon Greenberg, Adam Petrich, Hong Zebger-Gong, Dale Shuster and Pavan Vaddady**

### ARTICLES

29 Population pharmacokinetic-pharmacodynamic modeling of clopidogrel for dose regimen optimization based on CYP2C19 phenotypes: A proof of concept study
**Yun Seob Jung, Byung Hak Jin, Min Soo Park, Choon Ok Kim and Dongwoo Chae**

41 Integrating machine learning with pharmacokinetic models: Benefits of scientific machine learning in adding neural networks components to existing PK models
**Diego Valderrama, Ana Victoria Ponce-Bobadilla, Sven Mensing, Holger Fröhlich and Sven Stodtmann**

54 Cluster Gauss-Newton method for a quick approximation of profile likelihood: With application to physiologically-based pharmacokinetic models
**Yasunori Aoki and Yuichi Sugiyama**

68 Comparison of two-stage and joint TGI-OS modeling using data from six atezolizumab clinical studies in patients with metastatic non-small cell lung cancer
**Antonio Gonçalves, Mathilde Marchand, Phyllis Chan, Jin Y. Jin, Jérémie Guedj and René Bruno**

79 Applied physiologically-based pharmacokinetic modeling to assess uridine diphosphate-glucuronosyltransferase-mediated drug–drug interactions for Vericiguat
**Sebastian Frechen, Ibrahim Ince, André Dallmann, Michael Gerisch, Natalia A. Jungmann, Corina Becker, Maximilian Lobmeyer, Maria E. Trujillo, Shiyao Xu, Rolf Burghaus and Michaela Meyer**

93 Eliciting the antitumor immune response with a conditionally activated PD-L1 targeting antibody analyzed with a quantitative systems pharmacology model
**Alberto Ippolito, Hanwen Wang, Yu Zhang, Vahideh Vakil, Hojjat Bazzazi and Aleksander S. Popel**

106 Bridging population pharmacokinetic and semimechanistic absorption modeling of APX3330
**Larissa L. Silva, Robert E. Stratford, Richard Messmann, Mark R. Kelley and Sara K. Quinney**

118 Toward improved predictions of pharmacokinetics of transported drugs in hepatic impairment: Insights from the extended clearance model
**Flavia Storelli, Mayur K. Ladumor, Xiaomin Liang, Yurong Lai, Paresh P. Chothe, Osatohanmwen J. Enogieru, Raymond Evers and Jashvant D. Unadkat**

132 A convolution-based in vitro-in vivo correlation model for methylphenidate hydrochloride delayed-release and extended-release capsule
**Pawan Kumar Gupta, Bev Incledon, Jogarao V. S. Gobburu and Roberto Gomeni**

143 Tumor growth inhibition modeling in patients with second line biliary tract cancer and first line non-small cell lung cancer based on bintrafusp alfa trials
**Ana-Marija Milenković-Grišić, Nadia Terranova, Diane R. Mould, Yulia Vugmeyster, Thomas Mrowiec, Andreas Machl, Pascal Girard, Karthik Venkatakrishnan and Akash Khandelwal**

154 A multistate platform model for time-to-event endpoints in oncology clinical trials
**Chih-Wei Lin, Mario Nagase, Sameer Doshi and Sandeep Dutta**

168 Pediatric model-based dose optimization using a pooled exposure–response safety analysis for nivolumab and nivolumab plus ipilimumab combination in melanoma
**Shengnan Du, Yue Zhao, Zheyi Hu, Sihang Liu, Amit Roy, Jun Shen, Li Zhu and Lora Hamuro**

### CORRIGENDA

180 Correction to Network module-based drug repositioning for pulmonary arterial hypertension

181 Correction to: Consistent methods for fat free mass, creatinine clearance, and glomerular filtration rate to describe renal function from neonates to adults
